# The Combined Effect of Neuromuscular Electrical Stimulation and Insulin Therapy on Glycated Hemoglobin Concentrations, Lipid Profiles and Hemodynamic Parameters in Patients with Type-2-Diabetes and Hemiplegia Related to Ischemic Stroke: A Pilot Study

**DOI:** 10.3390/ijerph18073433

**Published:** 2021-03-26

**Authors:** Maja Rubinowicz-Zasada, Ewa Kucio, Anna Polak, Petr Stastny, Krzysztof Wierzbicki, Piotr Król, Cezary Kucio

**Affiliations:** 1 Doctoral School, The Jerzy Kukuczka Academy of Physical Education, 72A Mikolowska Str., 40-065 Katowice, Poland; mrubinowiczzasada@gmail.com; 2 Department of Neurology, Silesian Hospital, 4 Bielska Str, 43-400 Cieszyn, Poland; 3 Institute of Physiotherapy and Health Sciences, The Jerzy Kukuczka Academy of Physical Education, 72A Mikolowska Str., 40-065 Katowice, Poland; e.kucio@awf.katowice.pl (E.K.); p.krol@awf.katowice.pl (P.K.); c.kucio@awf.katowice.pl (C.K.); 4 Laboratory Physical Training Adaptation, Faculty of Physical Education and Sport, Charles University, Ovocny trh 5, 11636 Prague, Czech Republic; stastny@ftvs.cuni.cz; 5 Department of Medicine and Dentistry in Zabrze, The Silesian Medical University, 2 Traugutta Str., 40-055 Katowice, Poland; krzysztofwierzbicki@yahoo.com

**Keywords:** neuromuscular electrical stimulation (NMES), type 2 diabetes mellitus (T2DM), hemiplegia, ischemic stroke, physical therapy

## Abstract

Type-2-diabetes mellitus (T2DM) is a global problem of medical, social and economic consequences. Physical activity is a vital therapy in patients with T2DM, but some of them cannot exercise for various reasons. The purpose of our pilot study was to determine whether a combination of neuromuscular electrostimulation (NMES) and insulin therapy could improve the management of T2DM patients with hemiplegia caused by an ischemic stroke. Fifteen immobile patients with T2DM on insulin therapy were enrolled in the study. NMES was applied to their lower limbs for 60 min, 5 days a week, over a period of 12 weeks. The intervention caused statistically significant reductions in the blood concentrations of glycated hemoglobin, total cholesterol and low-density cholesterol in the participants. Furthermore, systolic and diastolic blood pressure levels were significantly lower. More randomized clinical trials are needed to accurately measure the effect of NMES on T2DM treatment and to determine whether it can be an alternative for physical activity for immobile patients with T2DM.

## 1. Introduction

Diabetes mellitus is a chronic metabolic disease symptomized by elevated blood glucose levels which develop as a result of decreased insulin secretion by the pancreas, insulin resistance or the joint effect of the two factors [1].

The global diabetes rate for adults rose from 4.7% in 1980 to 8.5% in 2014 [2]. It is estimated that by 2035 the number of diabetics worldwide will reach 592 million, entailing huge medical, economic and social costs [3]. As well as reducing diabetic patients’ quality of life and productivity, the disease frequently makes them avoid social events and leads to premature death. It is also a major cost for national healthcare budgets. The global cost of diabetes treatment is estimated at as much as USD 1.31 trillion (1.8% of global GDP) [4].

Most patients (~90%) are diagnosed with type 2 diabetes mellitus (T2DM) [5]. The disease is especially common among working age and elderly people. The 2018 statistics showed that although most Americans with T2DM were aged 45 years [6], the incidence of T2DM among people under 45 and even adolescents and children was rising fast [7,8,9].

T2DM patients are susceptible to many life-threatening microvascular and macrovascular complications including strokes, acute coronary events, blindness, amputations, kidney disease, infective endocarditis and heart failure. It is estimated that 1 in 12 deaths is caused by diabetes [10,11,12]. Patients with T2DM are at a much greater risk of premature death than the general population [12,13,14].

Metabolic disorders in patients with T2DM are treated with pharmaceuticals, diet, education and physical activity. Regular physical activity is reported to be effective in treating the early forms of T2DM and reducing the risk for cardiovascular complications [15,16]. Clinical trials [17,18] have shown that physical activity has many benefits for patients with T2DM, such as changes in metabolism, including increased insulin sensitivity, more efficient supply of insulin to the tissues and improved glucose metabolism. There is also evidence that it keeps glucose levels in the normal range when applied as part of a comprehensive diabetes treatment program [16]. Muscle glycogen usage during exercise increases glycogen synthase activity and carbohydrate production, which improves aerobic capacity and skeletal muscle exercise tolerance [18]. In the clinical trial by Strasser et al. [19], regular resistance exercises reduced blood glycated hemoglobin concentration (HbA_1C_), fat tissue and systolic blood pressure in participants affected by T2DM and at risk of T2DM.

However, some patients with T2DM and hemiplegia caused by a stroke, or motor system disorders, cannot exercise. The results of clinical studies indicate that physical exercise could be replaced in such cases by neuromuscular electrical stimulation (NMES). In the pilot clinical study by Wall et al. [20], NMES increased protein synthesis in the lower limbs of six men aged 65+ with T2DM and sarcopenia. Joubert et al. [21] reported considerably higher insulin sensitivity in T2DM patients who received lower limb NMES once a day for a week. In the clinical trial by Jabbour et al. [22], the concentrations of serum glucose measured in T2DM patients who received lower limb NMES (8 Hz) were significantly lower 60 and 120 min after the session than in the controls. Sinacore et al. [23] reported that lower limb NMES increased the number of type-2 muscle fibers in the study participants, as well as lowering their blood glucose concentration and improving insulin sensitivity.

The authors of all the cited studies presented evidence of the therapeutic usefulness of NMES for T2DM patients. The strength of this evidence is, however, limited for two reasons. Firstly, the studies are few and do not explain how exactly electrical stimulation improves the health of these patients. Secondly, they do not conclusively state whether NMES can be an alternative to physical exercise in the case of immobile T2DM patients. Therefore, more research in this area is necessary.

Given the above, this pilot trial was designed to determine whether a combination of NMES and insulin therapy would have an effect on glucose metabolism, serum cholesterol and triglycerides concentrations, cardiac hemodynamic parameters, and blood pressure in T2DM patients with hemiplegia caused by an ischemic stroke. Clinical studies of similar scope have not been conducted to date.

## 2. Materials and Methods

### 2.1. Study Design

The study was conducted in conformity with the ethical standards established by the 1964 Declaration of Helsinki and its later amendments. The ethical approval for the study was obtained from the Academy’s Bioethics Commission (No. 9/2012 of 10 October 2012). The trial was registered with the Australian-New Zealand Clinical Trials Registry (ANZCTRN 12618000486224).

### 2.2. Setting and Participants

The trial participants were selected from among patients treated in a neurological clinic (Silesian Hospital in Cieszyn, Poland) and assessed for eligibility by a physician against the following inclusion criteria: T2DM and hemiplegia from an ischemic stroke present for at least 3 months, a stabilized therapy with insulin for at least 6 months before the study, a stabilized pharmaceutical therapy with antihypertensive agents and antihyperlipidemic agents for at least 3 months before the study, body mass index (BMI) ≥ 25 kg/m^2^.

Excluded from the trial were patients with late complications of diabetes (retinopathy, neuropathy, diabetic nephropathy), cancer, acute and chronic diseases of the heart, liver and kidneys, acute myocarditis or pericarditis, uncontrolled hypertension, thromboembolism, nervous system disorders other than related to a stroke, patients with pacemakers, cardioverter defibrillators (ICD) or cardiac resynchronization therapy defibrillators (CRT-D), as well as patients ineligible for or unable to perform lower limb NMES.

All patients received written information about the purpose and design of the trial and that they could withdraw from it at any time without prejudice to their treatment.

### 2.3. Interventions

Patients’ characteristics were determined based on their medical records, standard interviews, and physical examinations. Patients with concomitant conditions had been on the same insulin therapy or drug therapy for at least 6 months and 3 months prior to the study, respectively (Table 1).

The NMES sessions were performed by the patients at their homes for 60 min, 5 days per week (excluding weekends), for a period of 12 weeks. During a session, the quadricep muscles were stimulated for 30 min and then the triceps muscles for another 30 min. Tetanic muscle contractions of 2 s duration alternated with rest periods of 4 s.

The patients used portable two-channel electrical stimulators Trio Stim (Mettler Electronics, CA Anaheim, USA), which delivered biphasic, rectangular pulses of 0.3 ms duration and a frequency of 35 Hz via self-adhesive silicone electrodes (5 × 10 cm, Dura Stick, Chatanooga, TN, USA) attached to the beginning and end of the muscle belly. Prior to the trial, a physiotherapist instructed the patients on how to operate the electrostimulators and then texted them daily to remind them about a session. The patients were free to call the physiotherapist to ask for assistance whenever needed. Moreover, the physiotherapist visited them weekly to see whether they performed NMES sessions as instructed, to check for side effects, and to provide additional guidance as needed.

To the authors’ knowledge, only two clinical trials investigating the effect of several weeks’ NMES on patients with T2DM have been published so far: a case series with 8 patients by Crowe et al. [24] and a randomized controlled cross-over study with 14 patients by Miyamoto et al. [25]. Crowe et al. [24] used 8 Hz NMES and stimulated patients’ quadricep muscles and calf muscles in both lower limbs for 40–60 min per day, 6 days per week, for 8 weeks. Miyamoto et al. [25] selected 5 Hz NMES and stimulated thigh and calf muscles during 40-min sessions performed 5 days per week over a period of 8 weeks. Low-frequency NMES (4 and 5 Hz) used in the studies only elicited single muscle contractions. In both studies, patients in the experimental and control groups were stabilized on therapy with standard drugs. After the intervention, Crowe et al. [24] reported a statistically significantly reduced concentration of HbA_1C_, an effect that Miyamoto et al. [25] did not observe. Moreover, Miyamoto et al. [25] did not find changes in the lipid levels in the experimental group, but the fasting glucose concentrations and percent body fat in the treated patients were statistically significantly lower and the concentrations of the brain-derived neurotrophic factor (BDNF) were statistically significantly higher. In neither of the studies was the effect of NMES on the hemodynamic parameters assessed. Because the purpose of our study was to determine the ability of the NMES to influence the concentration of HbA_1C_, lipid profiles, and hemodynamic parameters in T2DM patients, we used 35-Hz NMES eliciting incomplete, tetanic contractions of the muscles. NMES frequencies between 10 and 50 Hz (eliciting tetanic contractions) proved effective in our earlier study [26] and in the randomized clinical trials with patients with chronic cardiac deficiency conducted by other authors [27,28,29,30,31,32,33,34,35,36,37]. The authors of the studies who applied NMES for 3–12 weeks reported that it improved participants’ exercise capacity [26,28,29,30,31,32,34,36,37], metabolic and hemodynamic parameters, including peak VO_2_ [28,29,30,31,34,37], as well as reducing their diastolic blood pressure [29], and increasing LVEF (%) [26].

### 2.4. Measures

The blood concentration of HbA_1C_ was determined using a turbidimetric immunoinhibition method. The concentrations of triglycerides (TG), total cholesterol, high-density and low-density lipoprotein cholesterol fractions (HDL, LDL) were assessed by standard enzymatic-colorimetric methods. The laboratory tests were conducted at baseline and at week 12 using the Roche device and Cobas Integra Reagents (Roche Diagnostics, Warszawa, Poland). Participants’ blood pressure and the cardiac hemodynamic parameters (the left ventricular ejection fraction (LVEF) and the left ventricular end-systolic and end-diastolic dimensions (LVEDD, LVESD)) were measured by one- and two-dimensional echocardiography with the Vivid *7* ultrasound scanner (General Electric, Waukesha, WI, USA). Average daily systolic and diastolic blood pressures (BPs, BPd) were calculated after 24-h ambulatory blood pressure monitoring (24-h ABPM) with the Boso Tm 2430 device (Bosch and Sohn, Jungingen, Germany). 

## 3. Outcomes

### 3.1. Primary Outcomes

The main purpose of our pilot trial was to determine whether a combination of lower limb NMES and standard T2DM treatment would have an effect on glucose and lipid metabolism in patients with T2DM who could not exercise because of post-stroke hemiplegia. Accordingly, the primary outcome was blood concentrations of HbA_1C_, TG, total cholesterol, and HDL and LDL fractions after 12 weeks of intervention.

### 3.2. Secondary Outcomes

The trial also sought to establish whether a combination of lower limb NMES and standard T2DM treatment would improve the cardiac hemodynamic parameters and blood pressure in patients with T2DM and post-stroke hemiplegia. Hence, the secondary outcomes of the trial were the values of LVEF, LVEDD, LVESD, BPs, and BPd after 12 weeks of intervention.

## 4. Statistical Analysis

The distributions of participants’ characteristics at baseline and post-intervention and the distributions of the differences between measurements taken at the two time points were tested for skewness, kurtosis, and modality using the Shapiro–Wilk W-test. Because in all cases skewness and kurtosis were less than 4 and distributions were unimodal, the central value and dispersion were presented as means and standard deviations. The homogeneity of variance was evaluated by Leven’s test. When distributions were normal and the variances were not statistically significantly different, the parametric tests were used. Otherwise, the non-parametric tests were applied. The baseline and post-treatment values of patients’ body mass, BMI, blood concentrations of HbA1C, TGs, total cholesterol, LDL and HDL fractions, blood pressure (BPs, BPd) and cardiac hemodynamic parameters (LVEF, LVEDD, and LVESD) were compared by Wilcoxon’s test. The level of significance was set for all tests at *p* < 0.05. Statistical analyses were performed by a blinded person in Statistica v. 13.1 (StatSoft Polska Sp. z o.o. Krakow, Poland).

## 5. Results

**Figure 1:**
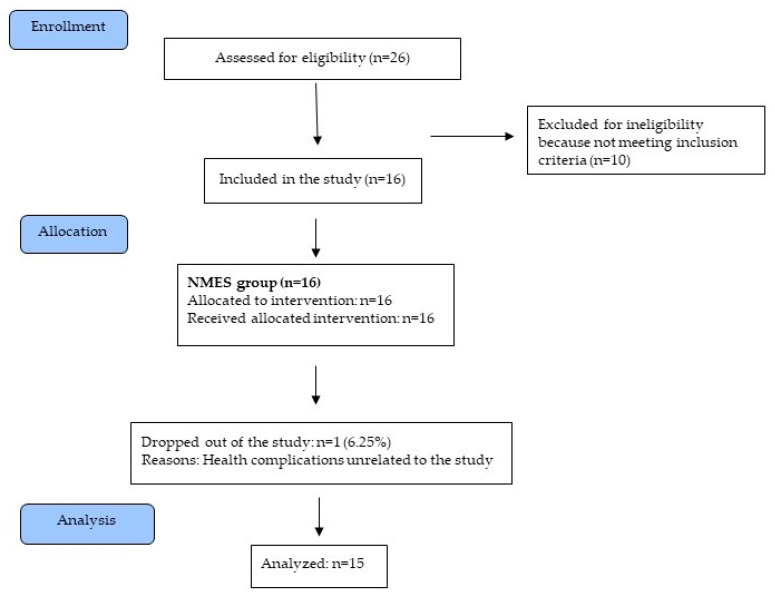
Diagram flow of the study.

Between 20 July 2015 and 29 January 2019, a group of 26 patients treated at a neurological clinic were assessed for their eligibility for the study. Of those, 16 met the inclusion criteria and were enrolled in the study. One patient did not complete the intervention for reasons unrelated to the study (Figure 1). Patients’ characteristics at baseline are shown in Table 2. The patients’ diet, daily routine, drug and insulin therapies were stabilized and did not require modification during the intervention period. NMES was not observed to cause any adverse effects that might require the discontinuation of intervention.

### 5.1. Primary Outcome

The mean concentration of HbA_1C_ decreased statistically significantly between baseline and week 12 (from 7.6% ± 1.28 to 7.2% ± 1.46; *p* = 0.0033). Significant decreases were also observed for total cholesterol (192.7 mg% ± 53.02 vs. 180.6 mg% ± 53.91; *p* = 0.0327) and LDL (110.1 mg% ± 44.7 vs. 98.5 mg% ± 40.33; *p* = 0.0011) (Table 3).

The concentrations of HDL and TG also decreased between baseline and week 12 (from 52.1 mg% ± 12.67 to 50.6 mg% ± 9.98 and from 148.6 mg% ± 62.79 to 142.3 mg% ± 77.15) but only in the second case the difference was statistically significant (*p* = 0.5188 vs. *p* = 0.8202). Neither patients’ body mass nor BMI was statistically different compared with their baseline values (*p* > 0.05) (Table 3).

### 5.2. Secondary Outcome

Mean BP decreased statistically significantly by week 12 (from 148.3 mmHg ± 18.58 to 137.2 mmHg ± 22.70 (*p* = 0.0231)), likewise mean BPd (from 80.5 mmHg ± 7.20 to 73.4 mmHg ± 11.90 (*p* = 0.0231) (Table 4.) Mean arterial pressure (MAP) was also lower (106.1 mmHg ± 17.69 vs. 101.6 mmHg ± 15.49) but the change was not statistically significant (*p* = 0.0995) (Table 4).

The values of the cardiac hemodynamic parameters (LVEF, LVESD, LVEDD) were higher post-intervention but the changes were not statistically significant—LVEF increased from 60.6% ± 6.99 to 61.1% ± SD 7.73 (*p* = 0.5541), LVESD from 28.5 mm ± 4.34 to 29.2 mm ± 5.28 (*p* = 0.0579), and LVEDD from 48.5 mm ± 4.56 to 50.5 mm ± SD 3.98 (*p* = 0.0555) (Table 4).

## 6. Discussion

### 6.1. Statement and Principal Findings

In our study, a 12-week intervention combining lower limb NMES, standard insulin and pharmacotherapy statistically and significantly reduced the blood levels of HbA_1C_, total cholesterol and LDL in T2DM patients with post-stroke hemiplegia, but their body weight and TG and HDL concentrations did not change. These results are preliminary, and more randomized clinical trials are needed to confirm whether NMES can replace physical exercise and improve glucose and cholesterol metabolism in immobile patients with T2DM.

Diastolic and systolic blood pressure measured at week 12 was also significantly lower than at baseline. Because diabetes is one of the risk factors for hypertensive heart disease, the ability of the NMES of large muscle groups to reduce blood pressure and improve the hemodynamic parameters in patients with T2DM should also be verified in randomized clinical trials. The result of this research would be especially useful in managing those of them who cannot exercise.

The values of LVEF, LVESD and LVEDD did not change significantly between baseline and week 12, echoing the results of our previous study with patients with chronic heart failure (CHF) [26]. It can, therefore, be presumed that the NMES protocol we used to elicit muscle contractions such as those induced by low-intensity aerobic exercise does not have an effect on the cardiac hemodynamic parameters, but this observation too should be further investigated in clinical research.

The results of clinical trials show that the effects of lower limb NMES and physical exercise are very comparable between patients with chronic obstructive pulmonary disease [38] and CHF [25,26,28,34,37]. NMES is reported to improve muscle blood flow [30], the activity of aerobic enzymes, and the function of vascular endothelium [30]. NMES has also been found to be able to reduce the concentration of pro-inflammatory cytokines [30] and improve the quality of life of persons with CHF [26,27,29,30,31,32,33,37] and pulmonary disorders [38]. According to Sinacore et al. [23], NMES is also capable of increasing the activity of type II muscle fibers.

The efficacy of NMES in the treatment of patients with T2DM is rarely assessed, but the results of the available studies are promising. The therapeutic effect of NMES was observed after single sessions [20,22], as well as after 1 week [21] and 8 weeks of NMES training [24,25].

Wall et al. [20] and Jabbour et al. [22] reported changes in the synthesis of muscle proteins [20] and metabolism [22] in patients with T2DM treated with NMES for 60 min. The authors of the studies used 60 Hz NMES and 8 Hz NMES eliciting tetanic muscle contractions and single muscle contractions, respectively. In both of them, stimulation intensity was set to produce strong and visible but comfortable muscle contractions.

Wall et al. [20] studied six sedentary men aged 70.3 ± 2.4 years, who had had T2DM for 7.8 ± 1.3 years on average. All patients were on oral glucose-lowering therapy, which was stabilized before the study. NMES (biphasic rectangular pulses of 0.5 ms, 60 Hz, a 3-s contraction/3-s relaxation time) was applied to the patients’ quadriceps muscle of one leg for 60 min, the other leg remaining untreated as a control. The patients also received continuous infusions of L-[ring-13C6]phenylalanine. Venous blood samples were taken before the NMES session, after the first 30 min, on completion of the session, and then every 30 min over the next 4 h. From the quadricep muscles of both legs, tissue samples were taken to assess the rate of muscle protein synthesis and the mRNA expression of genes involved in the regulation of muscle mass. The biopsy was performed before the NMES session and then 5, 120 and 240 min after it ended. Measurements showed that throughout the muscle regeneration period the rate of muscle protein synthesis was statistically and significantly higher for the stimulated limb than for the control limb (*p* < 0.01). The results reported by Wall et al. [20] are especially relevant to the treatment of older persons with T2DM, who are prone to atrophy due to prolonged physical inactivity.

Jabbour et al. [22] assessed the effect of a single, 60-min NMES session on the glucose profiles of 8 T2DM patients aged between 41 and 65. The glucose tolerance tests were carried out with and without NMES delivered to the participants’ knee extensors of both legs. The authors used low frequency (8 Hz) NMES with rectangular biphasic pulses of 0.2 ms duration. Three blood samples were collected: at rest, and then 60 and 120 min after consumption of a glucose load on the NMES and control days. Glucose concentrations on the NMES days were significantly lower (*p* < 0.01) than in the control conditions. Moreover, a significant positive correlation (r = 0.9, *p* < 0.01) was obtained between the intensity of NMES and changes in blood glucose. The authors concluded that 8 Hz NMES seemed suitable to enhance glucose uptake in persons with T2DM and that the effect might depend on stimulus intensity [22].

Joubert et al. [21] used NMES settings (similar to those we selected) in their study of 18 patients with T2DM (59.6 ± 8.3 years). Insulin sensitivity of the participants (treated with oral hypoglycemic agents and/or glucagon-like peptide 1 inhibitors) was compared between a single NMES session (*n* = 10) and a series of seven consecutive sessions (*n* = 8). In both cases, NMES (biphasic square pulses of 0.35 ms duration; 35 Hz) was applied to both quadriceps muscles for 25 min. The “on” and “off” times were fixed between 6 and 7 s. Similar to the cited studies, NMES intensity was set to elicit visible and strong but comfortable muscle contractions. Insulin sensitivity was evaluated by euglycemic hyperinsulinemic clamp before and after a single NMES session (group 1) and after a week of daily NMES training (group 2). Energy expenditure at baseline and during the NMES was evaluated by indirect calorimetry. Insulin sensitivity measured after the NMES was statistically significantly higher than at baseline only in group 2 (by 24.9 ± 35.8%; *p* = 0.009). The energy expenditure caused by the NMES was 1.42 ± 9.27 kcal/h, which was not significantly increased from the baseline. The authors reported that a 1-week training program of daily 25-min bi-quadricipital NMES sessions significantly improved insulin sensitivity in patients with T2DM treated with oral hypoglycemic agents and/or glucagon-like peptide 1 inhibitors [21].

There are only two clinical trials on the effect of several weeks’ treatment of T2DM patients with NMES published by Crowe et al. [24] and Miyamoto et al. [25]. The authors of both of them used low frequency NMES (<10 Hz) and stimulation intensity eliciting strong but comfortable muscle contractions (like in the aforementioned studies).

Crowe et al. [24] conducted a case series with eight generally healthy men with T2DM (mean age 53 ± 8 years; mean BMI 32 ± 5 kg/m^2^), who had been diagnosed with the disease within the past 5 years. NMES (5 Hz, biphasic pulses of 0.760–0.857 ms duration, with an interphase delay of 0.1 ms) was applied to both quadriceps muscles, hamstrings, gluteal and calf muscles. NMES sessions of 45–60 min were performed by the participants at their homes once a day, 6 days per week, over a period of 8 weeks. Measurements of HbA_1C_ levels at week 8 showed that they were statistically significantly higher than at baseline, by 0.8 ± 0.7% on average (7.4 ± 1.3% vs. 6.6 ± 1%; *p* = 0.01). The authors of the study concluded that aerobic NMES might be acceptable and have a beneficial effect on the HbA_1c_ of some men with T2DM, especially those of them who will not or cannot do adequate amounts of voluntary exercise. They also observed, however, that another randomized control trial was necessary for conclusive efficacy data to be obtained (Crowe et al. 2012).

Miyamoto et al. [25] designed their clinical study as a randomized controlled cross-over trial to examine the effect of NMES training on metabolic parameters and the levels of the plasma brain-derived neurotrophic factor (BDNF) and the insulin-like-growth factor (IGF-1) in patients with T2DM. The study participants were 14 individuals (63.2 ± 3.0 years, 76.1 ± 3.5 kg) with HbA_1C_ 7–9%, none of whom was treated with exogenous insulin injections. NMES was delivered to the gluteus maximus, quadriceps, hamstrings, hip abductor and adductor muscle groups, and dorsi- and plantar flexor muscle groups on each leg using monophasic exponential pulses (0.2 ms and 4 Hz). NMES sessions lasted 40 min and were performed by the participants at their homes, once a day, 5 days per week, over 8 weeks. The fasting glucose concentration measured at week 8 was statistically significantly lower (*p* = 0.041) than at baseline, by 3.3 ± 2.4% on average (9.0 ± 0.4 mmol/L vs. 8.5 ± 0.3 mmol/L). Percent body fat also decreased statistically significantly (*p* = 0.044), by an average of 29.4 ± 2.8% (from 27.3 ± 2.8% to 2.1 ± 2.0%). Its changes in the control period were not statistically significant (*p* > 0.05). Changes in the levels of HbA_1c_ and blood lipids were not significant in either the NMES period or the control period (*p* > 0.05). The BDNF levels increased significantly more in the NMES period than in the control period, by an average of 730.6 ± 289.0%. The application of NMES did not influence the blood concentration of IGF-1 (*p* > 0.05). Miyamoto et al. [25] concluded that an 8-week NMES training program could have a positive effect on the blood glucose concentration, percent body fat, and plasma BDNF levels in patients with T2DM, and that NMES training might prove to be an alternative exercise method for patients who might have difficulties in performing adequate voluntary exercise [25].

As promising as the results of ours and the other cited studies are, it is important to note that only one of the studies on patients with T2DM was a fully randomized clinical trial [25]. Therefore, the presented results need to be treated as preliminary and interpreted with caution [20,21,22,24]. Nevertheless, they are interesting enough to be verified by high-quality clinical trials.

According to the cited studies, 60 Hz and 35 Hz NMES can improve the synthesis of muscle proteins and, consequently, reduce the risk of muscle atrophy due to physical inactivity [20]. It is also reported to be able to increase insulin sensitivity (for this effect to be achieved a series of 7 NMES sessions of 25-min duration is necessary) [21]. Low-frequency NMES (4–8 Hz) has been found to reduce blood glucose concentration [22,25] and percent of body fat [25] and to increase the level of plasma brain-derived neurotrophic factor [25].

In our study, 12 weeks of treatment with 35 Hz NMES in conjunction with stabilized therapy with insulin and pharmacotherapy statistically and significantly reduced the concentrations of HbA_1C_ in patients with T2DM. The same effect was reported by Crowe et al. [24], who treated T2DM patients with 5 Hz NMES for 8 weeks. However, in the cross-over randomized clinical trial by Miyamoto et al. [25], significant changes in the concentration of HbA_1C_ in T2DM patients receiving 4 Hz NMES for 8 weeks were not observed. Therefore, more randomized clinical trials are necessary to assess the effect of different frequencies of NMES on HbA_1C_ concentration.

In our study, the post-intervention concentrations of total cholesterol and LDL were significantly lower than at baseline. This outcome is consistent with the findings reported by Gorgey et al. [39], who conducted a pilot clinical study of five patients with spinal cord injuries. NMES sessions were performed twice a week for a period 12 weeks (the authors did not state their duration) on the knee extensor muscles. The post-intervention measurements showed significantly lowered glucose curves and the concentrations of triglycerides and HDL in the participants. Gorgey et al. [39] used the same current waveform as we did (0.45 ms and 30 Hz pulses), which caused 5-s contractions and relaxations of the muscles. In Myamoto et al. [25], 4 Hz NMES sessions (performed daily for 40 min, 5 days per week, over a period of 8 weeks) did not have a statistically significant effect on the concentrations of triglycerides, total cholesterol and LDL and HDL in patients with T2DM. Therefore, more randomized, clinical studies are necessary to determine the effect of different NMES frequencies on metabolic risk factors for cardiovascular complications in patients with T2DM.

Type 2 diabetes mellitus leads to arterial hypertension and other heart diseases such as myocardial insufficiency. In our study, 12 weeks of NMES reduced participants’ systolic and diastolic blood pressure but the cardiac hemodynamic parameters did not change. It is probably the only clinical study evaluating the effect of NMES on blood pressure and cardiac hemodynamic parameters in persons with T2DM. There are, however, a number of randomized clinical trials with persons with chronic heart failure, which have demonstrated the ability of several-week NMES training to improve exercise capacity and metabolic and hemodynamic parameters [26,28,30,31,32,34,36,37], increase peak VO_2_ [28,29,30,31,34,37], reduce diastolic blood pressure [29], and increase heart rate [31,34] and LVEF (%) [26].

The NMES used in studies on people with CHF was similar to the one we used. During a session, the large groups of muscles of both lower limbs (mainly the thigh and calf muscles) were stimulated using 10–50 Hz current (mainly biphasic, with pulses of 0.2–1 ms duration), which caused strong but comfortable tetanic muscle contractions. The duration of contractions ranged from 2 to 20 s and the pauses between them were as long or twice longer. In most of the cited studies, NMES sessions were performed once day for 30 to 60 min (much less frequently for 2–4 h), 5–6 days per week, over a period of 3–12 weeks. A similar NMES protocol was used by Joubert et al. 2015, in which increased insulin sensitivity levels were observed in T2DM patients after 7 days of stimulation. The results of trials with CHF patients and the few trials with T2DM patients lead to a conclusion that >10 Hz NMES has a similar effect to physical exercise and induces positive metabolic and hemodynamic changes. This observation is of practical importance for managing T2DM.

Because of the still limited evidence that low frequency NMES (<10 Hz) can also be useful in treating T2DM, more randomized clinical trials are needed to obtain conclusive results.

Given that we did not observe any adverse effects of NMES over the 12-week intervention, nor have such effects been reported by the authors of studies with patients with T2DM and chronic heart failure, it seems that NMES is a procedure that can be safely performed by patients at their homes.

### 6.2. Strengths and Limitations.

The main limitation of our clinical trial is the relatively small size of the experimental group and the lack of a control group. At the same time, however, it is probably the first one to attempt to evaluate the usefulness of lower limb NMES in the treatment of patients with T2DM who cannot exercise as a result of hemiplegia caused by an ischemic stroke.

## 7. Conclusions

The increasing incidence of T2DM and strokes drives the search for new, effective therapies. Many randomized trials on patients with CHF who cannot exercise have presented evidence that NMES can be a substitute for physical activity. The same usefulness of NMES has been demonstrated by single clinical trials with patients with T2DM.

In our trial, 12 weeks of NMES at 35 Hz statistically and significantly reduced the blood concentrations of glycated hemoglobin, total and low-density cholesterol in T2DM patients with post-stroke hemiplegia, who had been on stabilized therapy with insulin, hypotensive drugs, and antihyperlipidemic agents. The changes suggest that NMES can be effective in improving lipid levels in T2DM patients who cannot exercise because of hemiplegia following an ischemic stroke. These tentative observations require confirmation by randomized clinical trials.

The ability of NMES to reduce systolic and diastolic blood pressure demonstrated by our trial is a strong argument for initiating clinical research on the effect of lower limb NMES in patients with T2DM and arterial hypertension who have suffered a stroke.

## Figures and Tables

**Table 1:** Concomitant conditions and pharmacological treatment (*n* = 15).

Concomitant Conditions	No. of Persons	Pharmacological Treatment	No. of Persons
Overweight	7	Beta adrenolytic drugs	13
Obesity	5	Angiotensin converting enzyme inhibitors	13
Generalized atherosclerosis	13	Calcium channel blockers	9
Arterial hypertension	15	Diuretics	11
Coronary disease	8	Nootropics	2
Secondary cardiomyopathy	3	AT1 receptor blockers	12
Trivial mitral and tricuspid regurgitation	3	Antithrombotic drugs	14
Post-infarction condition	2	Antihyperlipidemic agents	3

**Table 2:** Patients’ characteristics at baseline (*n* = 15).

Variable	NMES Group (*n* = 15) Mean ± SD
Gender: women/men	4/11
Age [years]	69.2 ± 7.76
BMI [kg/m^2^]	28.2 ± 2.98
Median duration of diabetes [years]	15.8 ± 4.57

NMES—neuromuscular electrostimulation; SD—standard deviation; BMI—body mass index.

**Table 3:** Mean metabolic parameters and BMI of patients before and after 12 weeks of intervention (*n* = 15).

Variable	NMES Group (*n* = 15)		Level of Significance (*p*)
	Baseline	Post-Intervention	
	Mean ± SD		
HbA_1C_ [%]	7.6 ± 1.28	7.2 ± 1.46	*p* = 0.0033
Total cholesterol [mg%]	192.7 ± 53.02	180.6 ± 53.91	*p* = 0.0327
LDL [mg%]	110.1 ± 44.70	98.5 ± 40.33	*p* = 0.0011
HDL [mg%]	52.1 ± 12.67	50.6 ± 9.98	*p* = 0.5188
TG [mg%]	148.6 ± 62.79	142.3 ± 77.15	*p* = 0.8202
Body mass [kg]	83.8 ± 11.6	83.6 ± 12.3	*p* = 0.9716
BMI [kg/m^2^]	27.7 ± 4.4	27.5 ± 4.6	*p* = 0.8416

NMES—neuromuscular electrostimulation; SD—standard deviation; BMI—body mass index; HbA_1C_—glycated hemoglobin; LDL—low-density lipoprotein cholesterol; HDL—high-density lipoprotein cholesterol; TG—triglycerides. The Wilcoxon’s test results.

**Table 4:** Mean daily arterial blood pressure and echocardiographic parameter values before and after 12 weeks of intervention (*n* = 15).

Variable	NMES Group (*n* = 15)		Level of Significance (*p*)
	Pre-Treatment	Post-Treatment	
	Mean (SD)		
BPs [mmHg]	148.3 ± 18.58	137.2 ± 22.70	*p* = 0.0231
BPd [mmHg]	80.5 ± 7.20	73.4 ± 11.90	*p* = 0.0231
MAP [mmHg]	106.1 ± 17.69	101.6 ± 15.49	*p* = 0.0995
LVEF [%]	60.6 ± 6.99	61.1 ± 7.73	*p* = 0.5541
LVESD [mm]	28.5 ± 4.34	29.2 ± 5.28	*p* = 0.0579
LVEDD [mm]	48.5 ± 4.56	50.5 ± 3.98	*p* = 0.0555

BPs—mean daily systolic blood pressure; BPd—mean daily diastolic blood pressure, MAP—mean arterial pressure; LVEF—left ventricular ejection fraction; LVEDD—left ventricular end-diastolic dimension; LVESD—left ventricular end-systolic dimension. The Wilcoxon’s test results.
